# Fractionation of Milligram Quantities of Gadolinium and Terbium Ions by Annular Free‐Flow Isotachophoresis

**DOI:** 10.1002/elps.70094

**Published:** 2026-04-11

**Authors:** Hannah Hallikainen, Xiaofeng Guo, Cornelius Ivory

**Affiliations:** ^1^ Voiland School of Chemical Engineering and Bioengineering Washington State University Pullman Washington USA; ^2^ Department of Chemistry Washington State University Pullman Washington USA

**Keywords:** electrophoresis, isotachophoresis, rare earth elements

## Abstract

The fractionation of milligram quantities of gadolinium (Gd) and terbium (Tb) ions is demonstrated using annular free‐flow isotachophoresis (ITP) with ammonium as the leading ion and hydronium as the terminating ion, and α‐hydroxyisobutyric acid (HIBA) and acetate as lanthanide complexing ligands. Buffer conditions were designed using numerical simulations in COMSOL Multiphysics, allowing for rapid screening of candidate buffer systems before laboratory experiments. The leading electrolyte contained 15 mM ammonium acetate, 5 mM acetic acid, and 3.5 mM HIBA at pH 4.9, while the terminating electrolyte consisted of 15 mM glutamic acid, 5 mM acetic acid, and 3.5 mM HIBA at pH 3.1. After 5 h of electrophoresis at 2 kV and at a mass loading of 7.077 mg Gd and 7.074 mg Tb, 84.08% of the Tb mass was recovered in the first 7 fractions removed from the separation column, and 86.51% of the Gd mass was recovered from the remaining 11 fractions. Overall recoveries of Gd and Tb were 95.07% and 99.13%, respectively.

## Abstract 2

The fractionation of milligram quantities of gadolinium and terbium ions is demonstrated using annular‐free flow isotachophoresis.

**Abbreviations**
HIBA: α‐hydroxyisobutyric acid
LE: leading electrolyte
npe: Nernst–Planck equations
REE: rare earth element
rpm: revolutions per minute
TE: terminating electrolyte

## 1 Introduction

The electrophoretic separation of lanthanides in α‐hydroxyisobutyric acid (HIBA) and acetate buffers has been previously demonstrated in microfluidic devices [1, 2] and capillaries [3]. Other complexing ligands, such as lactic acid [4] and malonic acid [5, 6], have also been utilized in small‐scale lanthanide separations by capillary electrophoresis. However, to the best of our knowledge, the electrophoretic separation of terbium and gadolinium ions at milligram scales by electrophoresis has not been previously reported. At these scales, lanthanide separations have generally relied on nonelectrophoretic separation methods, such as ion exchange chromatography, liquid–liquid extraction, and extraction chromatography [7].

In the work of Bottenus et al. [2], the gadolinium and terbium mass loading achieved on a microchip electrophoresis system using an acetate‐HIBA buffer system was on the order of 10^−5^ milligrams each. The mass loading of the separation demonstrated in this work is approximately 1 000 000 times larger in scale. It is more challenging to make a direct comparison to capillary separations of lanthanides, as mass loading is not frequently reported for such separations, but the present separation is between 4 [8] and 5 [9] orders of magnitude larger than the reported mass loadings described in capillary isotachophoresis (ITP) studies. Capillary separations of this type tend to be on the order of microgram or nanogram masses rather than milligrams. This significant increase in mass loading over capillary and microfluidic systems is enabled by the use of our annular free‐flow vortex‐stabilized electrophoresis instrument [10, 11, 12]. This apparatus was designed as a simplification of the recycle continuous flow electrophoresis (RCFE [13, 14]) device developed in the 1980s, which combined the recycle concept put forward in Bier's recycle isoelectric focusing (RIEF [15]) apparatus with a scalable version of the thin‐film free‐flow electrophoresis apparatus originally developed by Hannig and coworkers [16, 17]. Free flow electrophoresis has a long and storied history, which begins with Philpot [18, 19, 20] and is punctuated with advances in scale, both up [21, 22] and down [23, 24, 25, 26], as well as variations in design [27]. For a comprehensive review of this field, the following papers are recommended [28, 29].

Beyond electrophoretic separations, isolating terbium from gadolinium is also of interest for medical isotope production. Four terbium isotopes—Tb‐161 (β^−^ emitter), Tb‐149 (α emitter), Tb‐152 (positron emitter), and Tb‐155 (γ emitter) [30]—have been proposed for various clinical applications, with Tb‐161 being of particular interest due to its chemical and radiological similarities to established therapeutic isotope Lu‐177 [30]. The separation of Gd and Tb necessary for Tb‐161 production, however, is not directly comparable to the separation described here, as the ratio of gadolinium to terbium in an irradiated Gd‐160 target is very high [30]. For medical applications, this separation is generally accomplished by ion exchange chromatography [30, 31] or extraction chromatography [32, 33]. These approaches can achieve very high Tb‐161 purities, though often with reduced overall terbium and gadolinium recovery [30, 34]. One potential advantage of annular free‐flow electrophoresis over chromatographic methods is that at no stage in the separation are the lanthanides adsorbed onto any solid matrix. As a result, recovery of dissolved constituents in the electrophoretic system is accomplished simply by collecting the buffer solutions from the system.

In this work, the buffer solutions used for the separation of Gd and Tb were designed to be minimally toxic and compatible with boron nitride, acrylic, and other plastics and polymers that contact the buffers during electrophoresis.

## 2 Theory and Mathematics

A one‐dimensional simulation of isotachophoretic separation in the VortopHor system was developed using COMSOL Multiphysics 6.2 to aid in buffer design before experimental studies. Electrophoretic mobilities for acetate, HIBA, Gd^3^^+^, and Tb^3^^+^ complexes with acetate and HIBA, and complex stability constants were sourced from Hirokawa et al. [35]. Log β, the equilibrium constant describing the complexation of HIBA and acetate with Gd^3^^+^ and Tb^3^^+^, along with the electrophoretic mobilities of the resulting ions and complexes, is used to model their separation.

Acid dissociation constants (pKa1 = 2.16, pKa2 = 4.32) of glutamic acid at 25°C and an ionic strength of 10 mM, and the calculated limiting mobility (2.81E‐8 m^2^/V·s) were sourced from Včeláková et al. [36]. However, the inclusion of glutamic acid ultimately proved irrelevant as the pH of the terminating electrolyte (TE) at pH 3.1 was sufficiently high that cationic glutamic acid did not participate in this separation in the resulting COMSOL simulations. At pH 3.1, the cationic glutamic acid is present only as a minor species compared with the zwitterionic form.

While the leading electrolyte (LE) pH of 4.9 is high enough that the formation of anionic glutamate would be expected, glutamic acid was only present in the TE, and significant mixing of the LE and TE buffers was not anticipated during ITP. Because the anionic glutamate species does not meaningfully participate in the cationic separation, the mobility of the anionic species was set to the negative of the cationic mobility as a placeholder value—this was a deliberate simplification for the purposes of the 1D COMSOL simulation, as the anionic species were anticipated to migrate to the end of the separation column. The pKa of the amino group of glutamate was not implemented in this simulation because this simulation was intended for use in screening acidic buffer conditions only.

The mobility and stability constants for all other buffer components came from Pospíchal et al. [37]. The development of this COMSOL simulation was adapted from the methods described by Dixon et al. [38] and Cong et al. [1], with the key difference that, unlike Cong et al. [1], who implemented a 2D geometry, we followed Dixon et al. [38] in using a far less computationally intensive 1D geometry. Literature values used in the COMSOL simulation are shown in Table 1.

**TABLE 1:** COMSOL inputs, absolute ionic mobility, pK_a_, and Log β values at 25°C and an ionic strength of zero. Source: Hirokawa, et al., [35]

Ion	Electrophoretic mobility (m^2^/V·s)	Log β
Tb^3+^	6.9E‐08	—
TbHIBA^2+^	4.0E‐08	3.62
Tb(HIBA)_2_^+^	2.0E‐08	6.47
Tb(HIBA)_3_	0.0E+00	8.51
TbCH_3_COO^2+^	4.7E‐08	2.70
Tb(CH_3_COO)_2_^+^	2.4E‐08	4.50
Tb(CH_3_COO)_3_	0.0E+00	5.76
TbHIBA(CH_3_COO)^+^	2.2E‐08	5.90
Gd^3+^	7.0E‐08	—
GdHIBA^2+^	4.0E‐08	3.53
Gd(HIBA)_2_^+^	2.0E‐08	6.25
Gd(HIBA)_3_	0.0E+00	8.21
GdCH_3_COO^2+^	4.7E‐08	2.79
Gd(CH_3_COO)_2_^+^	2.4E‐08	4.65
Gd(CH_3_COO)_3_	0.0E+00	5.72
GdHIBA(CH_3_COO)^+^	2.2E‐08	5.84
(CH_3_COO)^−^	4.24E‐08	—
(HIBA)^−^	3.35E‐8	—

The simulation utilized the Nernst–Planck equations (npe) interface in COMSOL, following the methodology described by Dixon et al. [38]. The output of this simulation was a time‐concentration dataset for the modeled ions and complexes during electrophoresis across a one‐dimensional separation length with fixed concentrations at either end of the separation channel.

The literature values for the limiting molar ionic conductivity of an ion n (*µ_n_*) are related to the absolute ionic mobility (*µ_m,n_*) by the relation shown in Equation (1), in which *z_n_* is the ion charge number, and *F* is Faraday's constant.

(1) $$\mu_{n}=z_{n}Fu_{m,n}.$$

COMSOL's npe module requires electroneutrality, the constraint shown in Equation (2), where *N* is the total number of ions in the system. In the simulation developed, the acetate ion was selected for the purposes of COMSOL's electroneutrality constraint, but this does not imply that acetate is uncharged in this system. The acetate anion mobility and Log β values used for modeling the migration of acetate during electrophoresis are shown in Table 1.

(2) $$\sum_{n=1}^{N}z_{n}c_{n}=0.$$

The form of the Nernst‐Planck equation used in COMSOL's npe module [39] is shown in Equation (3), where Φ is the electric potential, *J* is the total number of reactions, *v* is the convective flow, and *D_n_* is the diffusion coefficient for ion *n*. The reaction term *R* is summed across all reactions *J* involving ion *n*. The current density is calculated as shown in Equation (4), under the constraint that electrical charge must be conserved in the system (Equation 5):

(3) $$\frac{\partial c_{n}}{\partial t}+\nabla \cdot \left(-D_{n}\nabla c_{n}-z_{n}u_{m},nFc_{n}\nabla \Phi \right)+v\cdot \nabla c_{n}=\sum_{j=1}^{J}R_{n},$$

(4) $$i=F\sum_{n=1}^{N}z_{n}\left(-D_{n}\nabla c_{n}-z_{n}u_{m},nFc_{n}\nabla \Phi \right),$$

(5) $$\nabla \cdot i=F\sum_{n=1}^{N}z_{n}\left(\sum_{j=1}^{J}R_{jn}=0\right).$$

The diffusion coefficient *D* for each complex was calculated using the Nernst‐Einstein equation (Equation 6), where *T_0_* is 298.15 K and *R_g_* is the universal gas constant (8.314 J mol^−1^ K^−1^). This procedure for estimating diffusion coefficients was adapted from Cong et al. [1].

(6) $$D_{n}=\frac{\mu_{n}R_{g}T_{0}}{Fz_{n}}.$$

Further explanation of the COMSOL npe module can be found in the COMSOL module documentation under “Governing Equations for the Nernst–Planck Formulation” [39]. The 1D separation column geometry used in the simulation has a length of 25.4 cm to match the real system. To ensure that solution volumes approximately match those of the real system, a cross‐sectional annulus area of 1.5959E‐4 m^2^ is used, along with a system dispersion coefficient of 2E‐8 m^2^/s.

## 3 Materials and Methods

All electrolytes were prepared in 18 MΩ deionized water (ELGA Labwater PURELAB flex 3). A 1 M acetic acid stock was prepared from glacial acetic acid (Fisher Chemical). The leading electrolyte (LE) was prepared by dissolving solid ammonium acetate (J.T. Baker) and HIBA (Alfa Aesar) in deionized water, and adding 1 M acetic acid. The terminating electrolyte (TE) was prepared by dissolving solid glutamic acid (Sigma‐Aldrich) and HIBA in deionized water and adding 1 M acetic acid. Solutions of terbium(III) acetate hydrate and gadolinium(III) acetate hydrate (Sigma‐Aldrich) were prepared by dissolution in a small volume of LE solution. The visual dye indicator was prepared by adding solid bromophenol blue (Bio‐Rad) and bovine serum albumin (Sigma‐Aldrich) to deionized water and was stored in a refrigerator at 2°C when not in use. Deionized water and buffers were not degassed before use.

Buffers were used as prepared, without further titration. The pH of the LE and TE, respectively, were 4.90 and 3.17 immediately after preparation. This separation requires a leading electrolyte (LE), a terminating electrolyte (TE), and a sample. The LE consists of 15 mM ammonium acetate, 5 mM acetic acid, and 3.5 mM HIBA, in which the ammonium cation acts as the leading cation and the acetate anion acts as the buffering counterion. The TE consists of 15 mM glutamic acid, 5 mM acetic acid, and 3.5 mM HIBA, in which the hydronium cation (originating from the acids) acts as the terminating cation and the acetate anion acts as the buffering counterion. In this system, the ammonium ion in the LE has a higher electrophoretic mobility than the various lanthanide–HIBA, lanthanide–acetate, and lanthanide–HIBA–acetate complexes, while the hydronium in the TE has a lower electrophoretic mobility than those complexes. While glutamic acid was included in the TE composition, at the TE pH (3.17), it is between its first and second pKas and does not function as a terminating ion. At this pH, the hydronium is the terminating ion. The use of hydronium as the terminating ion in isotachophoretic separations has been well established since the 1980s [40, 41]. Glutamic acid, acetic acid, and HIBA are partially dissociated and negatively charged in a pH range between the pH values of the LE and TE. Thus, anions of these acids cannot act as terminating ions of this cationic ITP system. The sample is 3 mL of 15 mM gadolinium acetate and 15 mM terbium acetate dissolved in LE, with the addition of 0.25 mL of a visual tracking dye—bromophenol blue bound to bovine serum albumin (1:1 molar ratio, 0.5 mM each).

The visual tracking dye is used to determine the approximate location of the ITP stack throughout the separation, because the lanthanide complexes and buffer solutions are not visibly distinguishable. In order to adjust the voltage and counterflow rate during electrophoresis, a visual tracking dye is needed to mark the approximate location of the sample bands. The bromophenol blue bound to bovine serum albumin has an electrophoretic mobility less than that of the gadolinium and terbium complexes, but greater than the effective mobility of the terminating hydronium cation. This results in a visible dye band forming between the lanthanide bands and the TE during electrophoresis. This use of bromophenol blue bound to bovine serum albumin as a visual marker in ITP was adapted from Harrison and Ivory [11] for use in an acidic buffer system.

The VortopHor (vortex‐stabilized electrophoresis) apparatus, developed by Cornelius Ivory [10], enables annular free‐flow electrophoresis in a 25.4 cm separation column with 20 mL separation volume. This apparatus enables significantly larger volume separations than conventional capillary electrophoresis systems. “Vortex stabilization” refers to the generation of stacked vortices within a fluid‐filled annulus between a boron‐nitride rotor and an acrylic stator. These vortices suppress dispersion and natural convection. Electrodes at either end of the separation column are placed in compartments separated from the separation column by permeable membranes (6000 molecular mass cut‐off dialysis membranes, Fisher Scientific). Buffer reservoirs provide fresh electrolyte for circulation through the electrode compartments and sweep out any gases generated at the electrodes.

For each experiment, the coolant circulator (VWR Refrigerated Circulator Model 1197) is set to 10°C, and the VortopHor rotor is turned on and set to 40 revolutions per minute (rpm). The buffer circulator reservoirs are filled with approximately 275 mL of LE and TE solutions, and the circulation pumps maintain a constant flow of liquid through the electrode compartments to ensure gases do not build up at the electrodes. Dialysis membranes separate each of the four electrode compartments (two at the top of the column, two at the bottom) from the separation column. The TE and LE are introduced into the separation column one at a time. First, the LE is injected through a syringe port at the bottom of the separation column, filling the bottom third of the separation column (8–9 mL). Then the TE is injected from a syringe port one third of the way up the column, and the top two‐thirds of the column are filled with TE (16–17 mL). Excess TE is pushed through the top of the column and through the waste collection lines at the top of the separation column. The 3.25 mL sample is injected through the same syringe port one third of the way up the column length, and the sample is slowly introduced into the separation column, followed by 0.5 mL LE to flush the injection line. At this point, the blue sample solution is located approximately in the center of the separation column, with the LE below it and the TE above.

After the sample has been introduced into the column, the high voltage power supply is turned on, and a 2 kV potential is applied across the column such that the cations migrate toward the electrodes at the bottom of the column. Simultaneously, fresh LE is delivered at 0.25 mL/min to the bottom of the separation column using a syringe pump. This maintains a counterflow to keep the sample suspended in the column; the formation of a stationary ITP stack in the VortopHor system has been previously reported by Harrison and Ivory [11]. Over the first 30–45 min of electrophoresis, the counterflow rate is slowly increased to 0.6 mL/min, and then held there until the voltage is shut off. Also, within the first 45 min, the visual tracking dye forms a thin, dark blue band at the sample‐TE interface. The location of this blue dye band is used to track the sample location and to determine when adjustments to the counterflow rate are necessary to hold the sample in a fixed position near the top of the separation column.

If the counterflow is insufficient, the lanthanide complexes will migrate to the bottom of the separation column, where they permeate through the dialysis membrane and are swept into the LE circulation reservoir. If the counterflow rate is too high or the voltage too low, the lanthanide complexes will be pushed out of the top of the separation column and into a waste collection container.

After the desired separation time had elapsed, the voltage and counterflow were stopped, but the rotor motion was maintained to slow the dispersion of the sample bands. Eighteen 1 mL fractions were collected from sampling ports along the length of the column stator. Fractions are collected immediately to minimize band dispersion, and all fractions were reserved for subsequent analysis. Collected fractions were diluted with 2% m/m HNO_3_ before analysis by inductively coupled plasma‐optical emission spectrometry (ICP‐OES) with an argon torch (Agilent Technologies 5100 ICP‐OES).

Unlike chromatographic techniques, this isotachophoretic method contains no solid‐phase materials that could adsorb or retain the target ions. All surfaces in contact with the sample solution are chemically inert and nonporous. Thus, the primary cause of reduced recovery is not adsorption, but overloading of the separation column, which allows excess lanthanides to migrate beyond the separation column into the electrode compartment, where they are swept into the buffer circulation reservoirs.

## 4 Results and Discussion

The COMSOL simulation allowed rapid screening of candidate buffer compositions before laboratory experiments with minimal computational resources. To achieve this, fluid flow and separation environment geometry were highly simplified to ensure that typical simulation run‐times were less than 1 h, with 108 027 total degrees of freedom and 27 dependent variables in the simulation. COMSOL simulations were run with a variety of buffer compositions using complex stability constants and mobility constants reported by Hirokawa et al. [35] and Pospíchal et al. [37]. Initially, a loading mass of 5 mg each of Gd and Tb was used for screening buffer compositions. It was found that a leading electrolyte consisting of 15 mM ammonium acetate, 5 mM acetic acid, and 3.5 mM HIBA, and a terminating buffer consisting of 15 mM glutamic acid, 5 mM acetic acid, and 3.5 mM HIBA produced the desired separation of Gd and Tb at a moderate pH range of 3–5. This buffer composition was used in all further experiments and simulations.

Three‐dimensional dispersion effects were accounted for in the model using a tunable dispersion coefficient, which can be refined by comparison to experimental data. The predicted separation profiles were generally in agreement with the laboratory experiments, although the simulations tended to overestimate the extent of separation and had a tendency to predict a “peak” shaped profile at high mass loadings (where experimental results yielded a broader “plateau” shaped profile). The accuracy of this simulation is dependent on the availability of accurate electrophoretic mobility and stability constant data for all buffer components. The simplicity of this simulation allows for low run times.

The overall recovery of Gd and Tb from the separation column is dependent on sample mass loading. As the lanthanide mass in the sample increases, total recovery in the separation column decreases while simultaneously lanthanide concentrations in the LE circulation reservoir increase. This suggests that excess lanthanides migrate toward the cathode, pass through the dialysis membranes separating the electrodes from the separation column, and are subsequently swept into the LE circulation reservoir.

Three mass loadings were tested: 9.17 mg (4.67 mg Gd/4.50 mg Tb), 14.13 mg (7.07 mg Gd/7.06 mg Tb), and 28.46 mg (14.15 mg Gd/14.30 mg Tb). The recovery at each mass loading is shown in Table 2. Mass recovery exceeded 99% at the lowest loading, dropped to 94.1% at 14.15 mg loading, and fell to 52.3% at 28.46 mg. Increasing sample mass loading also reduced the peak Gd and Tb concentrations after separation, with the peaks becoming broader and flatter at higher mass loadings. This trend is consistent with peak broadening, wherein the higher mass loading distributed the lanthanide ions over a longer column length, resulting in a “plateau” separation profile rather than a narrow “peak” profile.

**TABLE 2:** Total mass of Gd and Tb ions (rare earth element [REE] mass) loaded onto separation column and total mass recovery after 5 h electrophoresis.

REE mass loaded (mg)	REE mass recovered (mg)	Average recovery, %
9.17	9.17	>99.99
14.15	13.41	94.1
28.46	14.89	52.3

At the highest mass loading, the electrophoresis voltage had to be reduced (below 2 kV) due to the increased conductivity of the lanthanide‐rich sample solution and the overcurrent limit on the high voltage power supply used. Attempts to perform experiments with significantly higher mass loading (>100 mg rare earth element [REE] mass loading) could not be completed, even at lower voltages, due to operational limitations of the high voltage power supply and significant oscillations in the observed current during electrophoresis.

**FIGURE 1:**
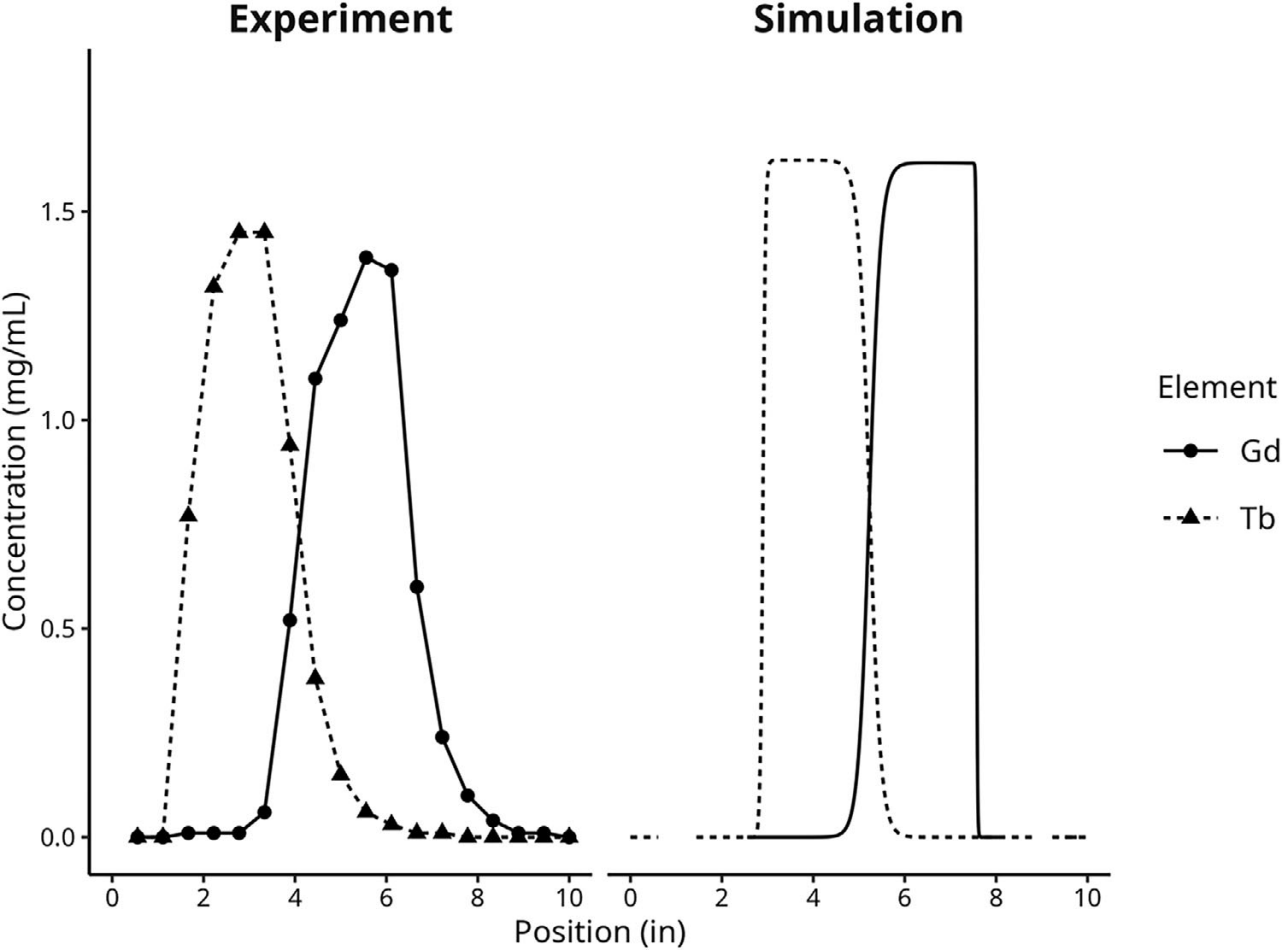
Left: Experimental Gd and Tb concentrations, Right: COMSOL simulation results, both with 7.077 mg Gd and 7.074 mg Tb initial loading and 5 h electrophoresis at 2 kV. Initial buffer composition and sample concentrations are the same between the experiment and simulation.

A total mass loading of 14.15 mg was selected for use in COMSOL simulations and time series experiments to maximize mass loading without significant loss of Gd and Tb recovery. Overall, the COMSOL simulation and experimental results match very closely when the Gd and Tb fractionation reaches equilibrium, as shown in Figure 1 at 5 h of electrophoresis for both the simulation and the experimental results. The experimental Gd and Tb peaks were somewhat broader than the simulated peaks, potentially due to dispersion during electrophoresis or resulting from the sampling procedure.

**FIGURE 2:**
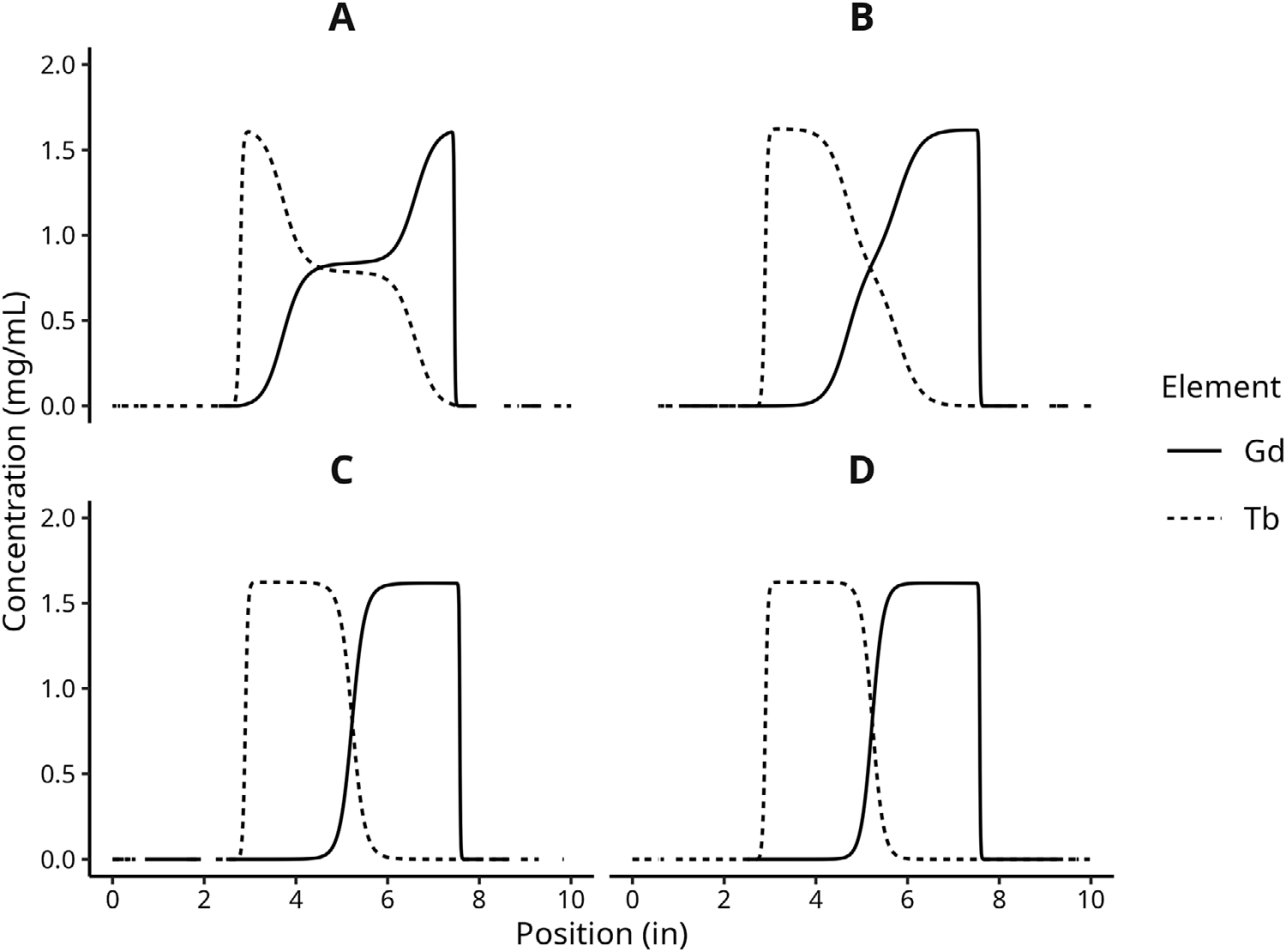
COMSOL simulation. (A) 1 h, (B) 2 h, (C) 3 h, (D) 4 h electrophoresis at 2 kV with initial loading of 7.077 mg Gd and 7.074 mg Tb.

**FIGURE 3:**
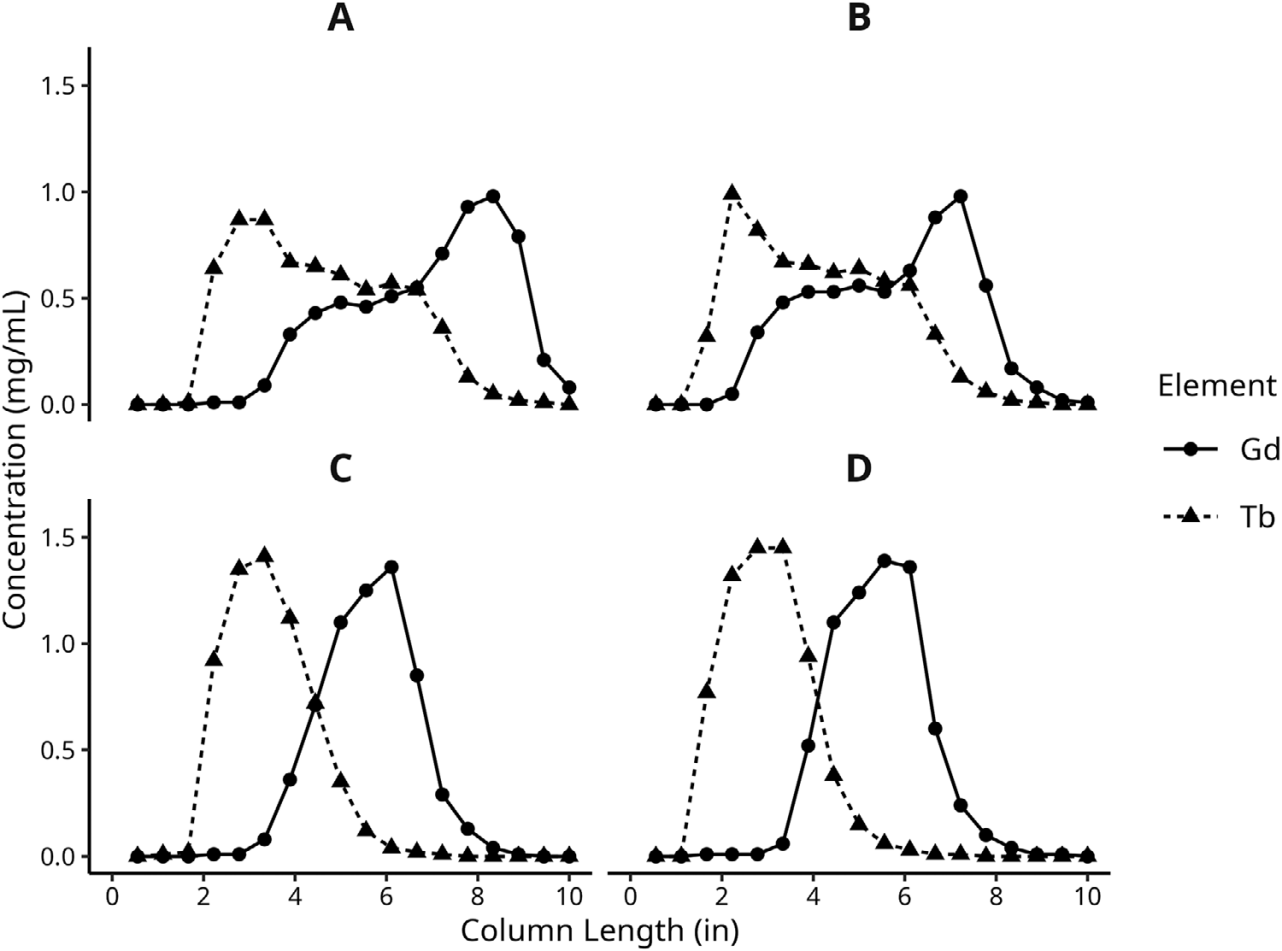
Experimental Gd and Tb concentrations across a 25.4 cm separation column length at (A) 2 h, (B) 3 h, (C) 4 h, (D) 5 h electrophoresis at 2 kV with initial loading of 7.077 mg Gd and 7.074 mg Tb.

The COMSOL simulation predicted that the separation between Gd and Tb would develop over the first 3–4 h of 2 kV electrophoresis, with the simulated concentrations across the separation column shown in Figure 2. Experimental results collected at 2–5 h of 2 kV electrophoresis, shown in Figure 3, produced separation profiles that were slightly more dispersed and approximately 1 h slower to develop than predicted in the simulation. This inconsistency in separation time may be corrected by adjusting the dispersion coefficient applied throughout the COMSOL simulation to more closely mimic the experimental system. Extending the electrophoresis time beyond 5 h in experiments yielded little improvement in the separation.

**FIGURE 4:**
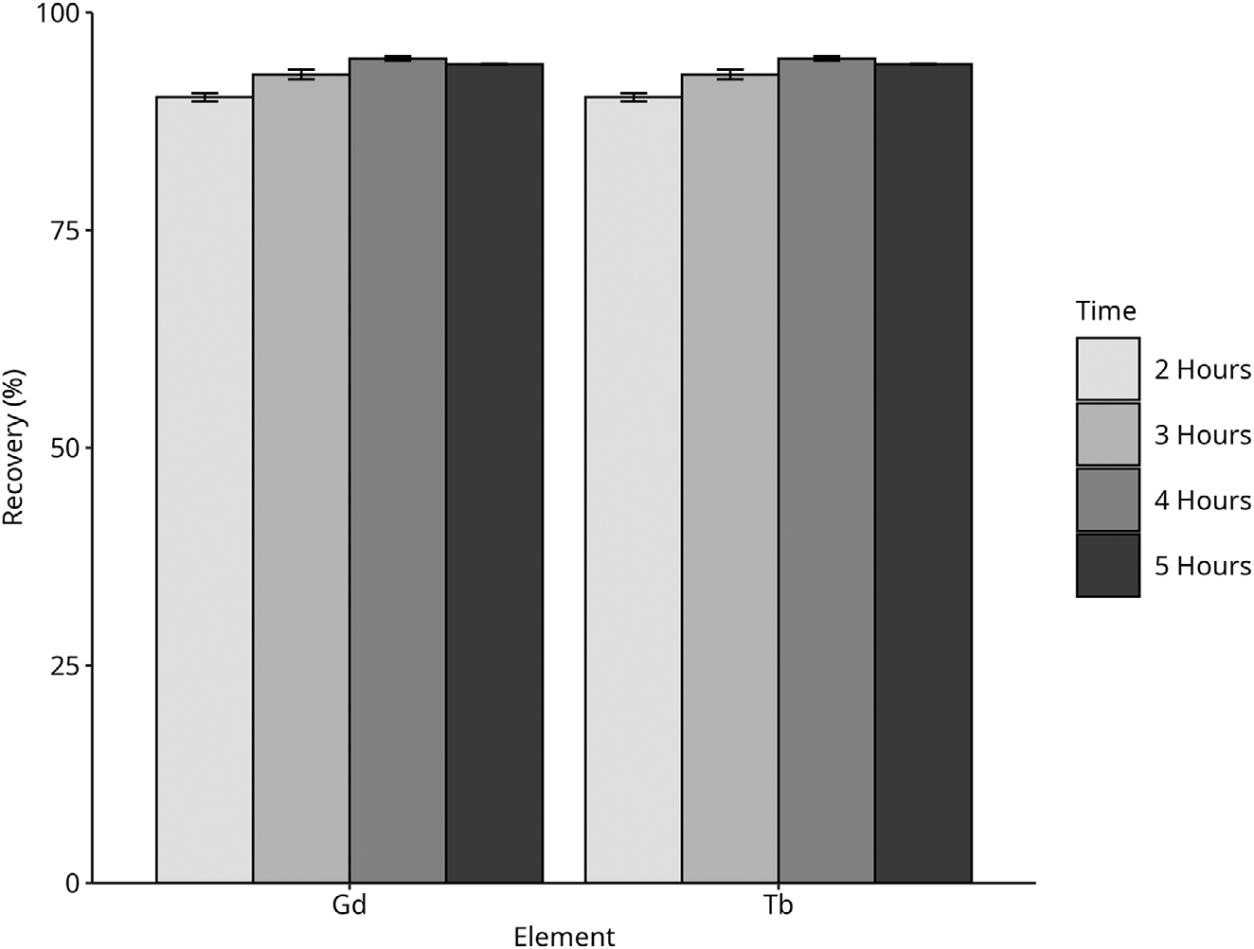
Overall mass recovery of Gd and Tb for 2–5 h experiments.

Slight variance in recoveries during the 2–5 h experiments was observed, shown in Figure 4, but overall mass recovery ranged from 90.28% to 94.73%, with the highest recovery obtained from the 4 h experiments. Differences in recovery between experiments may be due to peak broadening, leaving a small amount of Gd and Tb outside of the sampled range (see Figure 3A for an example of this), as well as small liquid volumes being left in the separation column annulus and syringe ports during sampling.

## 5 Conclusion

This work demonstrates a fractionation of adjacent lanthanide ions (Gd and Tb) at the milligram scale using an annular free‐flow electrophoresis apparatus, and the development of a simple COMSOL simulation for use in rapid testing of potential buffer compositions before laboratory experiments. While both the simulation and experiment reached very similar end states at 5 h, the differences in the speed of the separation could likely be corrected by fine‐tuning the diffusion and dispersion parameters in the COMSOL simulation. This fine‐tuning to more precisely match the annular free‐flow electrophoresis system used may be the subject of future work to improve the accuracy of this COMSOL simulation as a tool for rapidly screening buffer compositions for electrophoretic separations. Currently, no experimental data have been collected for electrophoresis times shorter than 2 h. Conducting shorter‐duration experiments may be of interest in future work to confirm whether the COMSOL simulation accurately captures the early stages of separation.

## Conflicts of Interest

The authors declare no conflicts of interest.

## Data Availability

Data are available from the corresponding author upon reasonable request.
